# Selective Extraction of Cannabinoid Compounds from Cannabis Seed Using Pressurized Hot Water Extraction

**DOI:** 10.3390/molecules25061335

**Published:** 2020-03-15

**Authors:** Yannick Nuapia, Hlanganani Tutu, Luke Chimuka, Ewa Cukrowska

**Affiliations:** Molecular Sciences Institute, School of Chemistry, University of Witwatersrand, Private Bag X3, Johannesburg, 2050, South Africa; Yannick.Nuapia@wits.ac.za (Y.N.); hlanganani.tutu@wits.ac.za (H.T.); luke.chimuka@wits.ac.za (L.C.)

**Keywords:** cannabinoid compounds, pressurized hot water extraction, response surface methodology

## Abstract

Phytochemicals of *Cannabis sativa* mainly for the use in the different industries are that of delta-9-tetrahydrocannabinol (THC) and cannabidiol (CBD). Pressurized hot water extraction (PHWE) is seen as an efficient, fast, green extraction technique for the removal of polar and semi-polar compounds from plant materials. The PHWE technique was applied to extract cannabinoid compounds from *Cannabis sativa* seed. Response surface methodology was used to investigate the influence of extraction time (5–60 min), extraction temperature (50–200 °C) and collector vessel temperature (25–200 °C) on the recovery of delta-9-tetrahydrocannabinol (THC), cannabinol (CBN), cannabidiol (CBD), cannabichromene (CBG) and cannabigerol (CBC) from *Cannabis sativa* seed by PHWE. The identification and semi quantification of cannabinoid compounds were determined using GCXGC-TOFMS. The results obtained from different extractions show that the amount of THC and CBN was drastically decreasing in the liquid extract when the temperature rose from 140 to 160 °C in the extraction cell and the collector′s vessel. The optimal conditions to extract more CBD, CBC, and CBG than THC and CBN were set at 150 °C, 160 °C and 45 min as extraction temperature, the temperature at collector vessel, and the extraction time, respectively. At this condition, the predicted and experimental ratio of THCt (THC + CBN)/CBDt (CBD + CBC+ CBG) was found to be 0.17 and 0.18, respectively. Therefore, PHWE can be seen as an alternative to the classic extraction approach as the efficiency is higher and it is environmentally friendly.

## 1. Introduction

*Cannabis L Sativa* is a plant belonging to the family of Cannabaccae and grows to around 2–5 m in height [1]. The plant is considered dioecious since it contains both male and female parts [2]. It exhibits several psychoactive and medicinal activities. The use of Cannabis has been recorded throughout history in many industries, such as food, cosmetics, paper, clothing, and pharmaceutical [3,4]. The phytochemicals within the plant have gained interest overtime for its medicinal purposes, mainly for its use against cancer-inducing effects [4,5].

Up today, there are above 500 compounds within the *Cannabis L Sativa* plant which are known, the most interesting is a set of compounds known as cannabinoids, there are about 70 different cannabinoids which are unique to the *Cannabis L Sativa* plant [5,6]. Among these, the most psychoactive compound being delta-9-tetrahydrocannabinol (THC), the metabolic breakdown of the compound, which is cannabinol (CBN), is still psychoactive, but less than that of THC. The second most abundant compounds are that of cannabidiol (CBD), cannabichromene (CBG) and cannabigerol (CBC). These compounds are synthesized from cannabinoid acids [1,7]. CBD, as explained, is one of the main anticancer components found in the *Cannabis L Sativa* plant. It is mainly found in the form of cannabidiolic acid (CBDA) and changes to CBD through decarboxylation [7,8].

The problem with the use of cannabis in pharmaceuticals and other endeavors is the presence of the THC and CBN in the plant, which is considered the more psychoactive and toxic for a human [8]. It can also affect the brain of young adults under the age of 21 years, and it has shown to affect school performance by reducing IQ [9]. Hence, various extraction techniques were applied to isolate or suppress the psychoactive compounds from the extract [1,10].

The extraction of cannabinoid compounds from cannabis has attracted the attention of many researchers [2,5,11,12]. Several extraction techniques of cannabinoid compounds from *Cannabis L Sativa* rely on the use of of conventional extraction approaches such as distillation, solvent extraction, Soxhlet, maceration, and sonication, however, these techniques are time-consuming and use an important volume of organic solvent with toxicity for environmental and human well-being [13,14]. Hence, the development of modern extraction techniques like ultrasound-assisted extraction (UAE), supercritical extraction (SFE) and pressurized hot water extraction are emerging as an alternative [15,16]. These techniques present a significant advantage over conventional methods. These advantages are less degradation, elimination of additional sample clean up, reduction in organic solvent consumption, concentration steps before chromatographic analysis, selectivity, improvement in kinetics, and extraction efficiency and ease of automation [17,18]. In other hand, UAE is much faster and low cost than the traditional extraction techniques. Nevertheless, the efficiency of the technique depends on the nature of the target analytes. Besides, it should be kept in mind that the distribution of the ultrasonic wave in the vessel is not uniform. It is limited to the vicinity of the ultrasound probe, making its application on a big scale a difficult task. SFE was more selective than the UAE method. It provides the advantages of high diffusivity and low viscosity. The main disadvantage is a high running cost [5,12,19,20].

The alternative to these techniques is pressurized hot water extraction (PHWE). It is considered a green extraction method. Instead of CO_2,_ water is used under its supercritical form as a solvent that exhibits the same solvability properties as methanol and ethanol [21]. Pressurized hot water extraction has proved to be an excellent approach for the recovery of the polar and semi-polar bioactive compounds from plant materials [21,22]. PHWE technique is based on the use of high temperature and pressure to keep the water in the supercritical fluid form during the entire extraction process [23]. The technique has attracted attention due to its benefits as compared to other conventional and non-conventional extraction approaches [21]. PHWE has been widely used for the extraction of phenolic compounds [24]. In this technique, temperature is very important; it directly affects extraction efficiency and the mass transfer during the extraction process [25,26].

Since THC and CBN are the main psychoactive compounds from cannabis plant, several researcher have developed techniques for a high selectivity extraction of non-psychoactive compounds over the psychoactive one. For example, Romano and Hazekamp [20] have used an infusion extraction approach to recover more CBD than THC selectively. The final extract obtained by them was heated up to evaporate THC and keep CBD in the solution. Perrotin-Brunel et al. [27], and Grijo et al. [28] have explored the solubility properties of CO_2_ and pressure for an efficient extraction of CBD. The results showed that the solubility of cannabinoids compounds increase with the increase of the pressure. To our knowledge, there are no studies that have applied PHWE for selective extraction of cannabinoid compounds from *Cannabis L Sativa* seeds. Therefore, the present study includes a pressurized hot water extraction process that yields a formulated cannabinoid nutraceutical. The PHWE technique was applied to cannabis seed in order to extract more CBD and lessen the THC and CBN, reducing the psycho-activity of cannabis products.

## 2. Materials and Methods

### 2.1. Chemicals and Reagents

Chemicals used in this study were of analytical grade. Methanol was used as a solvent and purchased from Sigma-Aldrich (Johannesburg, South Africa).

### 2.2. Plant Material

One species of cannabis was subjected to extraction and analysis. Seed samples of *Cannabis L Sativa* were collected from farmers in Gauteng province in August 2019. Prior to processing, seeds were dried at 50 °C for 4 h and the water content was found to be 5.5–7.9% weight. The dried samples were crushed to powder and kept in a sealed container until extraction.

### 2.3. Response Surface Methodology

The influence of extraction temperature (80–200 °C), the temperature at collector vessel (25–200 °C) and extraction time (15–60 min) on the selective recovery of cannabinoid compounds were investigated using response surface methodology (RSM). Full factorial design, comprising of 30 experiment run with three center point replicates, was created in MODDE Pro (Sartorius Stedim Biotech, Malmö, Sweden), to assess the recovery of THC, CBN, CBD, CBG, and CBC from the *Cannabis sativa* seeds. During the optimization process, the pressure and flow rate were kept constant at 105 bar and 0.5 mL min ^−1^, respectively, according to our previous work [24]. The partial least square regression was applied to evaluate the fitting of the model and response surface. The adequacy of the models was evaluated by the R^2^ and Q^2^ values (where R^2^ shows the model fit and Q^2^ shows an estimate of the future prediction precision). The F-test was used to assess the significance of the coefficients of regression. The modeling was done with a quadratic model like linear, squared, and interaction terms.

### 2.4. Pressurized Hot Water Extraction

**Figure 1:**
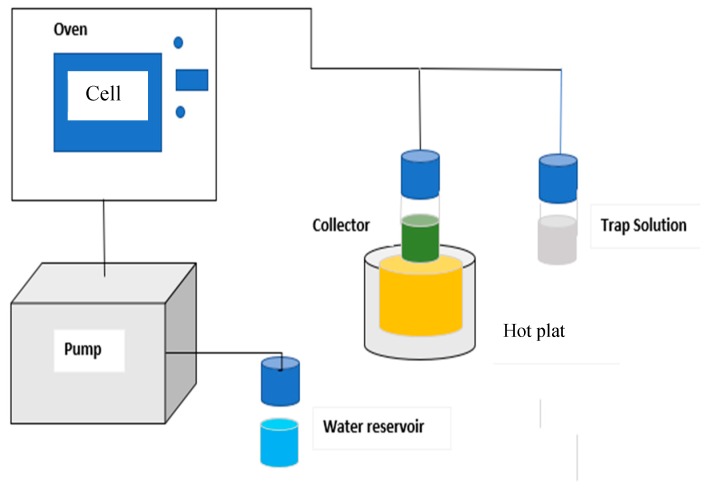
Pressurized hot water extraction setup with showing trap solution and oil heat bath for collector.

Pressurized hot water extraction instrument was constructed in the laboratory and equipped with an extraction cell, gas chromatography (GC) oven for controlling the extraction temperature and time, and a collector vessel (Figure 1). This was in accordance with the method described by [24] with some modifications. For each run, a mass of 5 g of *Cannabis sativa* seed powder was filled into the PHWE extraction cell. Before each extraction, the cell loaded with the powder was pre-heated at a specific extraction temperature for 10 min. The dynamic extractions were done under various extraction conditions as specified by the CCD matrix (Table 1). The approach used in the pressurized hot water extraction for cannabinoid compounds is to extraction.

The responses were expressed in percentage relative peak area, which is the peak area of each compound over the total peak area in the chromatogram; these percentages are generated by the instrument. During the extraction process, the collector′s vessel was heated at different temperatures in order to investigate the effect of these temperature variations on the removal of cannabinoid compounds in the obtained liquid extract.

### 2.5. GCXGC-TOFMS Method

A LECO 2D GCXGC chromatography with a time-of-flight mass spectrometer (LECO, Johannesburg, South Africa) was used for the identification and analysis of cannabinoid compounds from *Cannabis sativa* seed. This experimental GCXGC-QTOF-MS/MS method developed by Marrotti et al. [29] was used with some modification. The separation was carried out in a 30 m × 0.25 mm × 0.25 μm BPX5 column (SGE Analytical, Johannesburg, South Africa). The injection was carried out in spit mode (ratio 20:1). The carrier gas was helium (99.999% purity) and was used as at a flow rate of 1 mL min^−1^. The oven temperature was set at 100 °C (for 2 min) and increased to 280 °C with rate of 15 °C min^−1^. Temperatures applied were 300 °C for injector, 250 °C for transfer line, 250 °C for ion source, and 150 °C for quadrupole. Data were acquired in the full scan mode with mass ranging from 45–600 amu. The total ion chromatograms (TIC) were integrated. Raw data (*m*/*z*) generated by UHPSFC were processed using the ChromaTOF software version 4.5.1. (LECO Corp, St. Joseph, MI, USA).

### 2.6. Quality Assurance

Deionized water was used throughout the study. Glassware was properly cleaned, and all the reagents were of analytical grade. All extractions were done in triplicates. All measurements were inter-day repeatability measurements taken over a week. In addition, the optimum extraction condition was partially validated for repeatability and reproducibility using relative peak areas.

### 2.7. Model Fitting and Predictive Efficiency

The model fitting and predictive efficiency between the experimental and predicted data at optimum extraction conditions were investigated by using the absolute average deviation (AAD), root mean square error (RMSE), mean absolute error (MAE), standard error of prediction (SEP), model predictive error (MPE), and chi-square statistic (χ^2^), and correlation coefficients (R^2^). The equations used to calculate these factors are described in Table 2.

## 3. Results and Discussion

### 3.1. Identification of Cannabinoid Compounds

**Figure 2:**
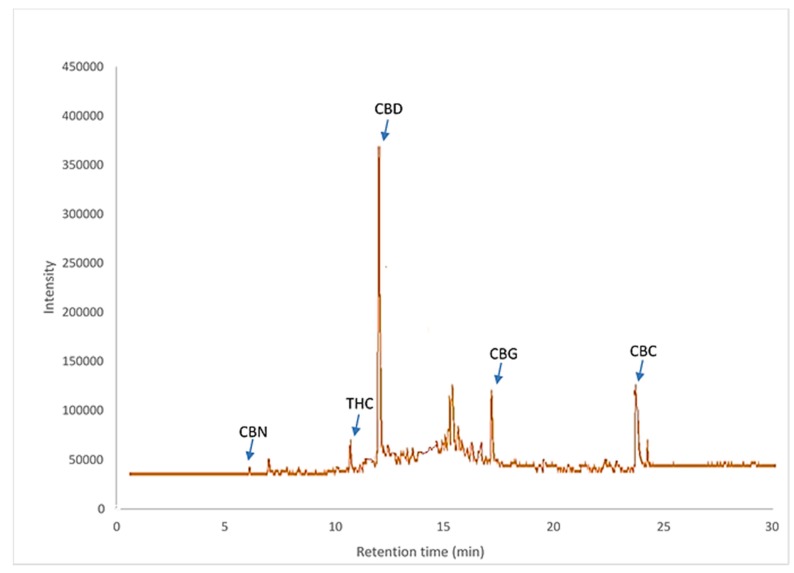
Chromatogram (TIC) of cannabis extract at optimum condition.

**Figure 3:**
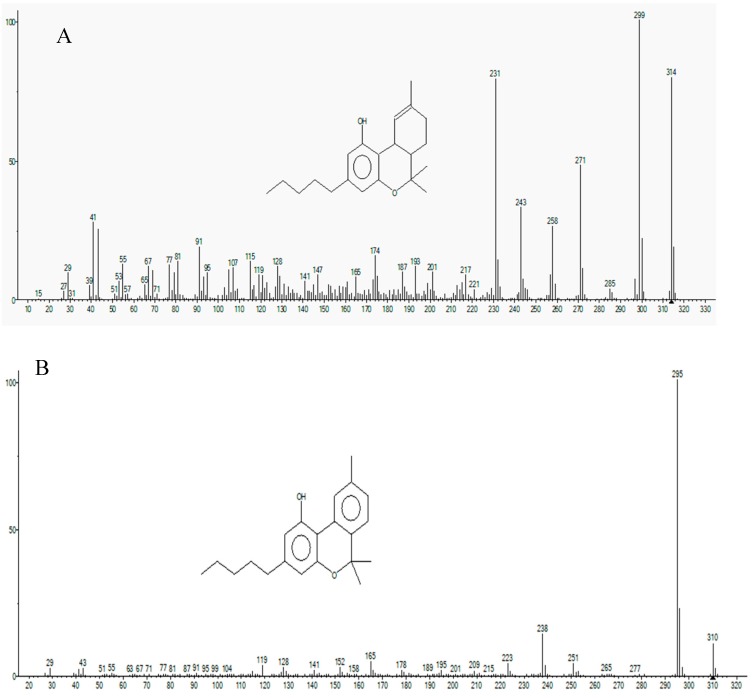
Fragmentation pattern of THC (**A**) and CBN (**B**).

**Figure 4:**
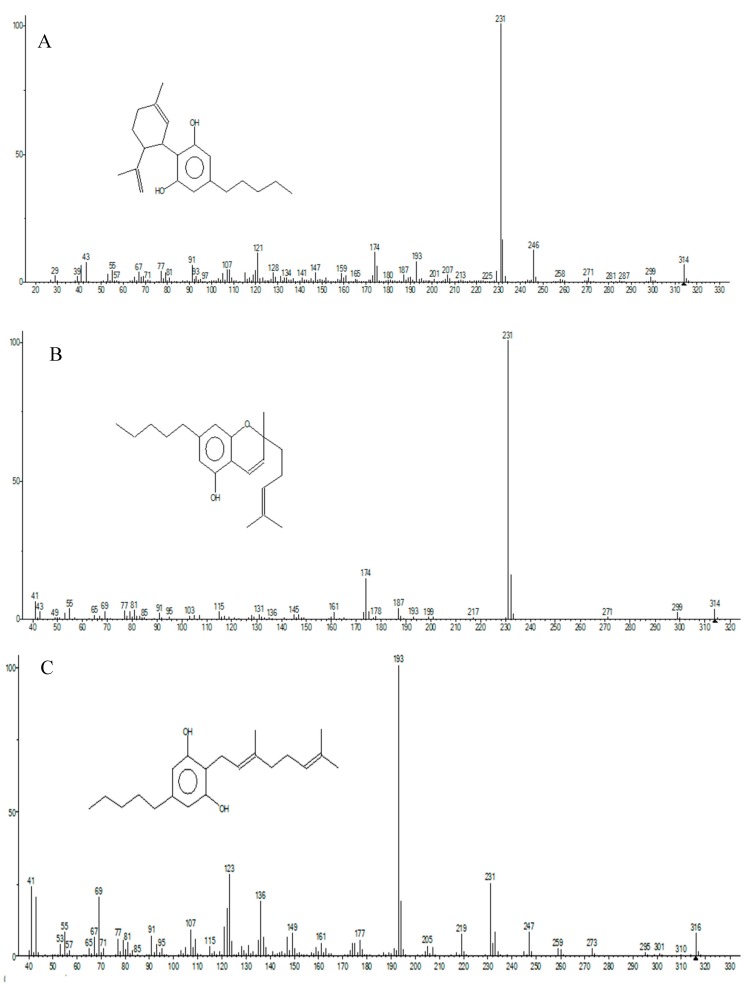
Fragmentation pattern of CBD (**A**), CBC (**B**) and CBG (**C**).

Cannabinoid compounds were identified using GC–TOF/MS. The chromatogram and fragmentation patterns are presented in Figure 2, Figure 3 and Figure 4. Five cannabinoid compounds such as THC, CBN, CBD, CBC, and CBG were identified from the plant extracts. Five cannabinoid compounds have been identified in the cannabis extracts. Figure 2 shows a chromatogram obtained for a cannabis extract.

The mass spectrum fragmentation obtained for THC, CBN, CBD, CBC, and CBG were compared with the fragmentation provided by the Leco database library.

The fragment partens were compared to the Leco database (NIST library). The mass spectrum fragmentation obtained for THC, CBN, CBD, CBC, and CBG were compared with the fragmentation provided by the Leco database library. The obtained fragmentation pattern is presented in Figure 3 and Figure 4. The similarity for all the fragmentation patterns for the targeted compounds with the library databe ranged from 80–98%.

### 3.2. Experimental Design: Response Surface Methodology

**Figure 5:**
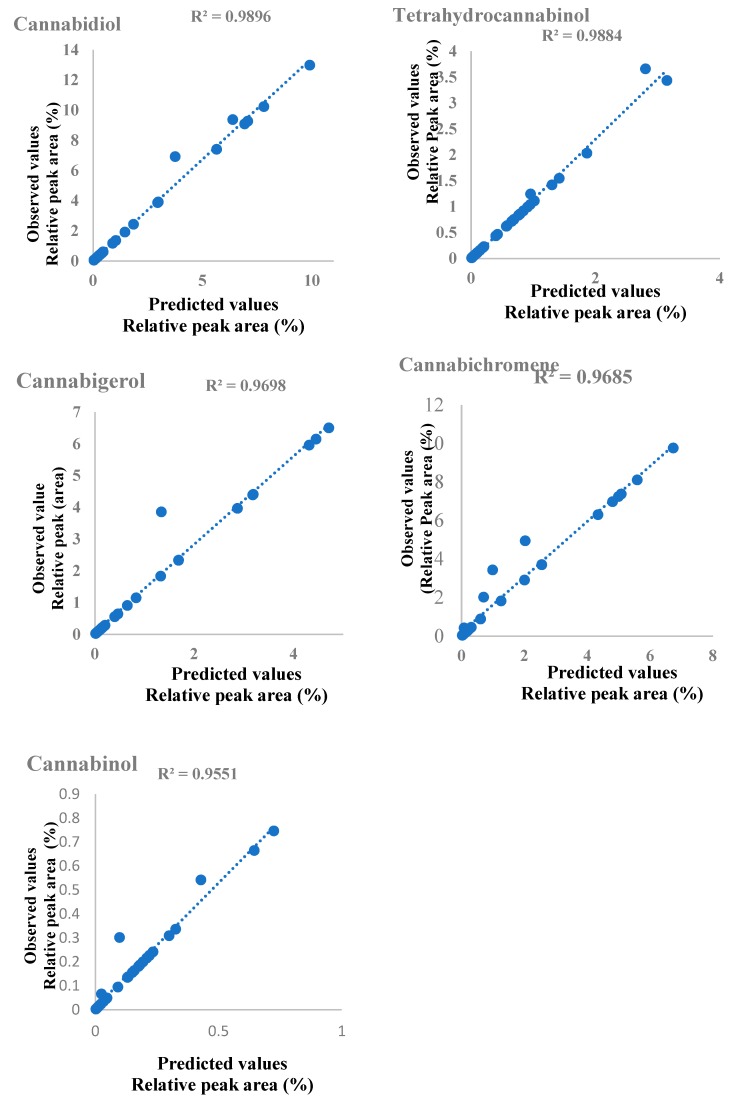
Predicted vs. observed values plot.

**Figure 6:**
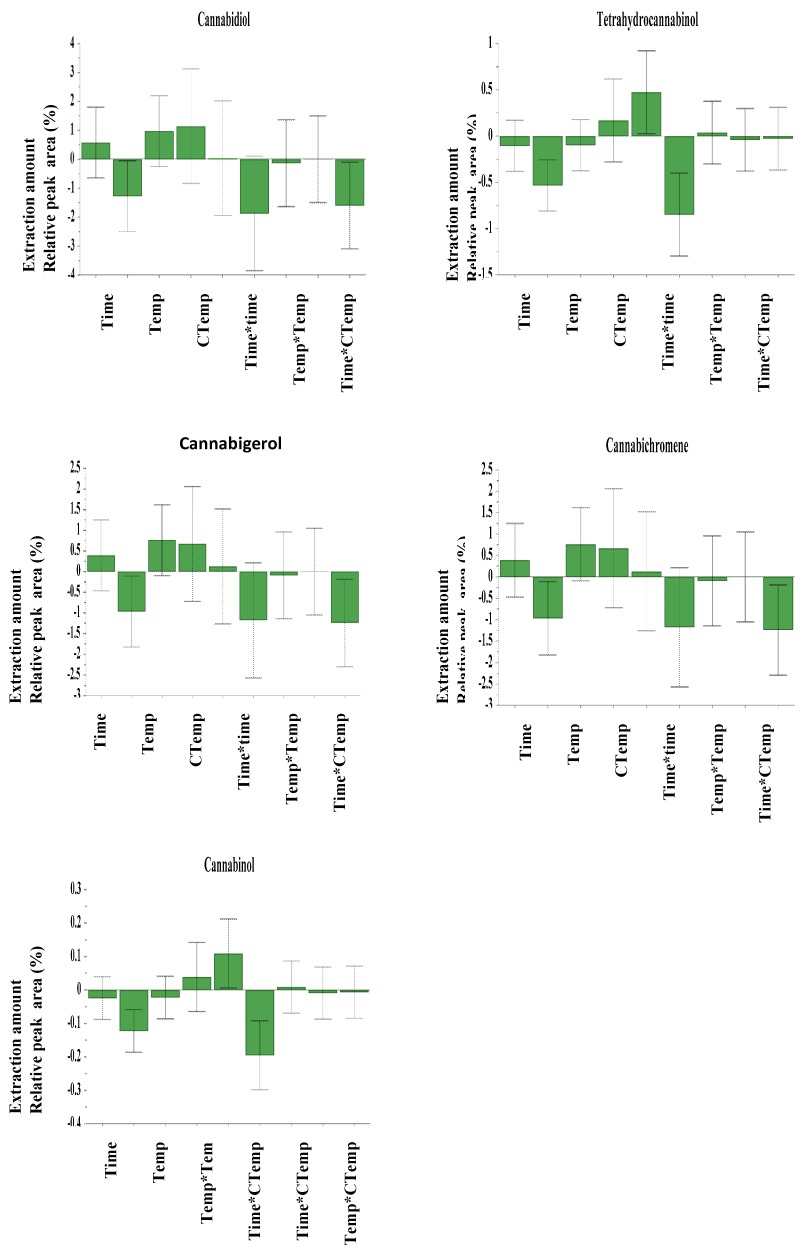
Coefficient plot.

Collector vessel temperature, extraction time and temperature were chosen for optimization since they are the main parameters that can influence the selectivity extraction of cannabinoid compounds from *Cannabis sativa* seed, and thereby, also the recovery efficiency. The extracted amounts of cannabinoid compounds expressed in relative peak area (%) at different experiments of the design are summarized in Table 1. The model was created and calculated by fitting using partial least square regression. The fitted model showed a total described variance range from 90%–95% (R^2^ = 0.96–0.99) and a cross-validated predictability ranged from 95–99% (Q^2^ = 0.95 – 0.99), where R^2^ shows the model fit and Q^2^ shows an estimation of the future prediction and precision [24]. The linearity of the predicted vs. observed values plot (Figure 5) highlighted the validity of the model and its capability to predict the best condition of the extraction within the range of the design. The coefficients plot (Figure 6) reveals that collector vessel temperature, extraction temperature and time have a significant negative influence on the extracted amount of Tetrahydrocannabinol (THC) and Cannabinol (CBN) with a p-value of 0.003, whereas extraction time has shown a significant positive influence on the amount extraction of cannabidiol (CBD), cannabichromene (CBG) and cannabigerol (CBC).

**Figure 7:**
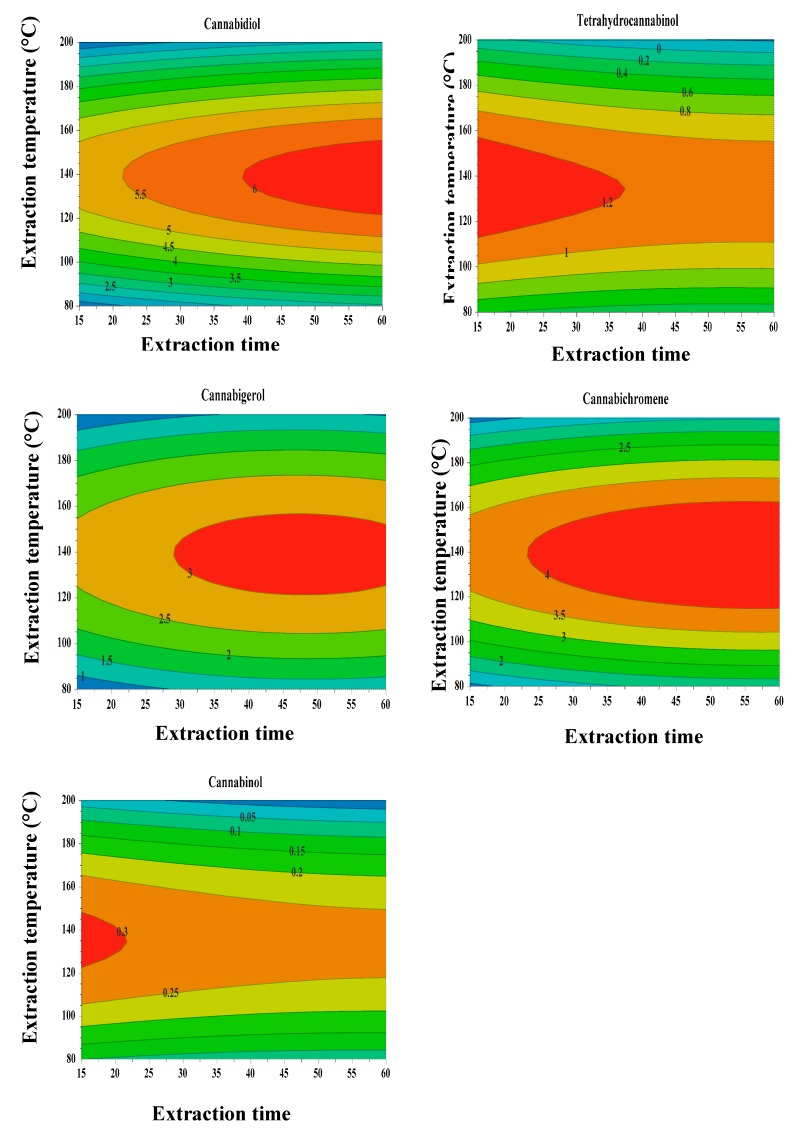
Counter plot.

**Figure 8:**
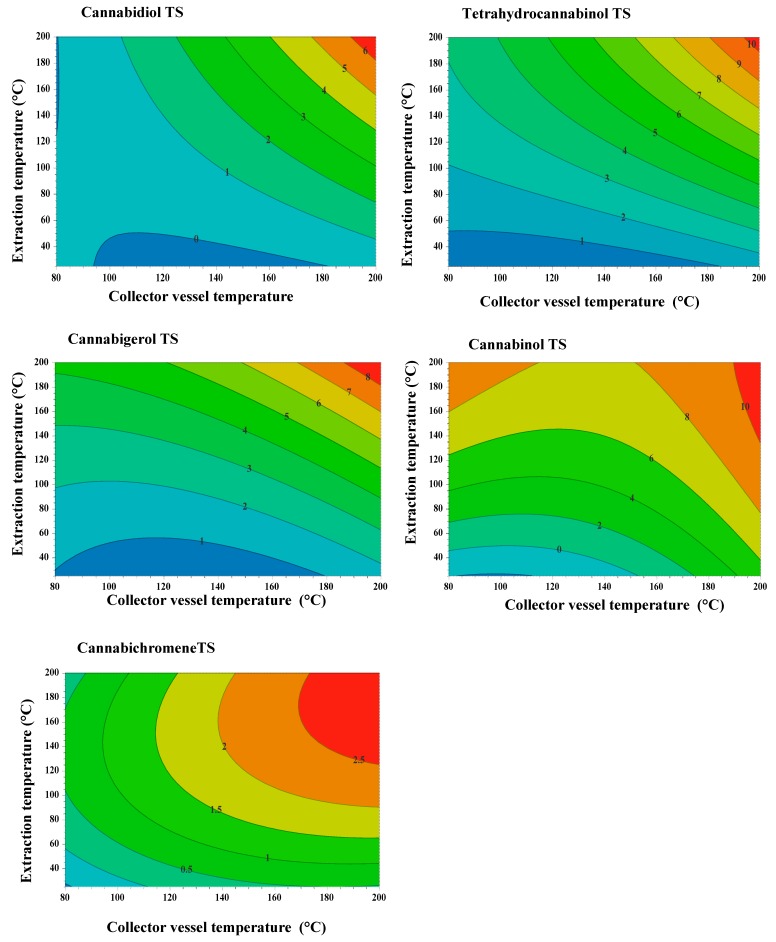
Counter plot: (TS = trap solution ).

The response surface described by a counter-plot in Figure 7 shows the direct and interaction impact of extraction time and temperature on the extracted amount of cannabinoid compounds from cannabis seeds. Increasing the extraction temperature and collector vessel temperature from 140 to 160 °C for 60 min decreased the extracted amount of THC and CBN, while the amount of CBD, CBC and CBG are raised in the extract. This is probably due to the vaporization of the THC and CBN portion at these temperatures, which ranged in temperature in liquid extract and increased the solubility of CBD, CBC and CBG in the liquid extract [20,29]. The increase of temperature from 140 to 160 °C in the extraction cell increased the solubility of the cannabinoids compounds. The collected extract was heated in the extraction cell at the temperature range from 140 to 160 °C. At this point, a portion of THC and CBN was evaporated from the extract, resulting in the final product having lower levels of THC and CBN and high amounts of CBD, CBC and CBG. Since the PHWE was equipped with the trapping system, which contained methanol, the evaporated THC and CBN were captured in this solvent. This resulted in the increase of levels of CBN and THC in the trapping solution (Figure 8). However, a further increase in temperature above 160 °C removed both psychoactive and non-psychoactive compounds from the extract.

### 3.3. Model Fitting and Predictive Efficiency Analysis

The response surface methodology approach was investigated for its predictive performance and estimation capabilities. The statistical factors including AAD (%), RMSE, MAE, SEP (%), MPE (%), and chi-square (χ^2^) have been assessed by equations (described in Table 2. The obtained results of the statistical analysis are shown in Table 3.

The results showed that RSM has a higher ability to investigate the interaction of the main parameters involving the extraction of cannabinoid compounds from cannabis seed by PHWE. Partial least square regression analysis was carried out between the response (THC, CBD, CBC, CBG, and CBN) values predicted by RSM models with their corresponding experimental value. Also, The RSM model predictions are much closer to the line of perfect prediction as presented in Figure 1. Also, this higher predictive accuracy of the RSM can be attributed to its ability to approximate the linearity for a small data set. All statistical error parameters are very low, proving good fitting of the model.

### 3.4. Universal Extraction Condition of an Extract rich in CBD, CBC, and CBG

It is clear that the optimal pressurised hot water extraction condition would be the one for an extract with lower THC and CBN, and high CBD, CBC and CBG. For systematic optimization, a desirability function was used to find the setting of extraction temperature, collector vessel temperature and extraction time where a high amount of CBD, CBC and CBG can be extracted. Accordingly, the best condition was found at 150 °C, 160 °C and 45 min for extraction temperature, collector vessel temperature and extraction time, respectively. The amounts of cannabinoid compounds expected to be extracted under these conditions using the predicted model are shown in Table 4.

The universal extraction conditions were applied to the *Cannabis sativa* seed to determine the exact extracted amounts. It was observed that the RSM predicted values for the extraction of cannabinoid compounds were within the standard deviation of the experimental values. Also, the repeatability and reproducibility ranged from 89.45 ± 2.87 to 92.61 ± 4.5% and 89.70 ± 5.60 to 92.56 ± 2.31%, respectively. The ratio between the THCt (THC + CBN) and CBDt (CBD + CBC+ CBG) was found to be 0.18, which means that the final extract at the optimal condition has more CBD, CBC and CBG than THC and CBN.

## 4. Conclusions

The present study was conducted in order to investigate the influence of extraction temperature, extraction time and collector vessel temperature on the pressurized hot water extraction of cannabinoid compounds from *Cannabis sativa* seed. Response surface methodology was performed as an optimization tool. The evaluation of different error statistical parameters such as AAD, RMSE, MAE, MPE, χ^2^, and R^2^ have shown that RSM is an excellent statistical tool in terms of prediction and estimation capabilities. The optimization of the PHWE process by RSM predicted that the ratio between THCt (THC + CBN) portion and CBDt (CBD + CBC+ CBG) portion was 0.17 under optimum conditions of 150 °C, 160 °C and 45 min for extraction temperature, collector vessel temperature and extraction time, respectively, which means that the final extract at the optimal condition has higher amounts of CBD, CBC and CBG than THC and CBN. To conclude, the optimization of PHWE using the RSM approach provides an effective guideline for an extraction process that yields a formulated cannabinoid nutraceutical. The PHWE technique has produced *Cannabis sativa* extract with high amounts of non-psychoactive compounds and lower levels of THC and CBN.

## Figures and Tables

**Table 1:** Experimental design with the response of independent factors (relative peak area).

Run Order	Extraction Time (min)	Extraction Temp. (°C)	Collector Temp.(°C)	THC (%)	CBD (%)	CBC (%)	CBG (%)	CBN (%)
19	15	80	25	0.5700	0.2989	0.2033	0.1345	0.1308
25	37.5	80	25	0.7900	0.4589	0.3121	0.2065	0.1812
7	60	80	25	0.9584	0.8978	0.6105	0.4040	0.2204
1	15	80	113	0.3989	0.2890	0.1965	0.1301	0.0917
4	37.5	80	113	0.9088	0.4556	0.3098	0.2050	0.2091
9	60	80	113	0.4278	0.8926	0.6070	0.4017	0.0984
21	15	80	200	0.2078	0.2699	0.1835	0.1215	0.0478
5	37.5	80	200	0.1889	0.3174	0.2158	0.1428	0.0435
18	60	80	200	0.1089	0.3878	0.2637	0.1745	0.0251
12	15	140	25	3.1504	1.4589	0.9921	0.6565	0.7246
10	37.5	140	25	2.8062	2.9819	2.0277	1.3419	0.6454
29	60	140	25	1.8639	3.7532	2.5522	1.6889	0.4287
15	15	140	113	1.4193	6.3856	4.3422	2.8735	0.3264
16	37.5	140	113	1.3032	7.0702	4.8077	3.1816	0.2997
20	60	140	113	1.0187	9.9046	6.7351	4.4571	0.2343
6	15	140	200	0.9537	1.0347	0.7036	0.4656	0.2194
2	37.5	140	200	0.6547	1.8502	1.2581	0.8326	0.1506
30	60	140	200	0.5809	2.9506	2.0064	1.3278	0.1336
11	37.5	140	113	0.7637	6.9309	4.9930	4.3189	0.1757
27	37.5	140	113	0.8428	5.6506	5.0824	3.1928	0.1938
14	37.5	140	113	0.6904	7.8078	5.5893	4.7135	0.1588
17	15	200	25	0.1444	0.1229	0.0836	0.0553	0.0332
23	37.5	200	25	0.1015	0.3353	0.2280	0.1509	0.0233
22	60	200	25	0.0927	0.4536	0.3085	0.2041	0.0213
8	15	200	113	0.0702	0.1099	0.0747	0.0495	0.0162
26	37.5	200	113	0.0497	0.1653	0.1124	0.0744	0.0114
3	60	200	113	0.0312	0.2221	0.1510	0.0999	0.0072
13	15	200	200	0.0201	0.0384	0.0261	0.0173	0.0046
24	37.5	200	200	0.0148	0.0407	0.0277	0.0183	0.0034
28	60	200	200	0.0105	0.0751	0.0511	0.0338	0.0024

**Table 2:** Equations of error functions.

Error	Equation
Absolute average deviation	AAD=[∑i=1n((|Yiexp−Yical|)Yiexp)n]
Root mean square error	RMSE $=\sqrt{\frac{\sum_{i=1}^{n}{\left(Y_{i,e}-Y_{i,p}\right)}^{2}}{n}}$
Mean absolute error	$\mathrm{MAE}=\frac{i}{n}\overset{n}{\underset{i=1}{\sum }}\left|Y_{i,e}-Y_{i,p}\right|$
Standard error of prediction (%)	SEP$\left(\%\right)=\frac{\mathrm{RMSE}}{Y_{i,e}}\times 100$
Model predictive error (%)	MPE$\left(\%\right)=\frac{100}{n}\overset{n}{\underset{i=1}{\sum }}\left|\frac{Y_{i,e}-Y_{i,p}}{Y_{i,p}}\right|$
Chi-square (χ^2^)	$\chi^{2}=\overset{n}{\underset{i=1}{\sum }}\frac{Y_{i,p}-Y_{i,e}}{Y_{i,p}}$
Correlation R^2^	$R^{2}=\frac{\sum_{i=1}^{n}\left(Y_{i,p}-Y_{i,e}\right)}{\sum_{i=1}^{n}{\left(Y_{i,p}-Y_{i,e}\right)}^{2}}$

AAD: Absolute average deviation, RMSE: Root mean square error, MAE: Mean absolute error, SEP: Standard error of prediction, MPE: Model predictive error, χ^2^: Chi-square statistic, R^2^: Correlation coefficients.

**Table 3:** Statistical results of response surface methodology model fitting and predictive efficiency analysis.

Parameter	THC	CBD	CBC	CBG	CBN
AAD	0.0067	0.0036	0.0021	0.0018	0.0014
RMSE	0.0400	0.0300	0.0600	0.0800	0.0700
MAE	0.0050	0.0020	0.0010	0.0020	0.0010
SEP	0.0120	0.0210	0.0140	0.0160	0.0190
MPE	0.0290	0.0160	0.0080	0.0090	0.0040
χ^2^	0.0005	0.0002	0.0005	0.0003	0.0009

**Table 4:** Predicted and experimental extraction values of each compound under universal conditions.

Responses	Predicted Values	Experimental Value (*n* = 3)	Repeatability (%RSD)	Reproducibility (%RSD)
THC	2.00	2.03 ± 0.20	90.23 ± 2.45	89.78 ± 2.34
CBD	5.40	5.60 ± 0.35	89.45 ± 2.87	91.34 ± 1.32
CBC	4.50	5.00 ± 0.60	92.45 ± 3.71	89.70 ± 5.60
CBG	3.50	4.10 ± 0.80	90.56 ± 3.56	92.56 ± 2.31
CBN	2.90	0.34 ± 0.09	92.61 ± 4.5	90.78 ± 2.19
THCt ^*^/CBDt ^*^	0.17	0.18	-	-

THCt = THC + CBN; CBDt = CBD + CBC+ CBG;Predicted and experimental values are express in relative peak area (%).
